# Molecular and *in vivo* Functions of the CDK8 and CDK19 Kinase Modules

**DOI:** 10.3389/fcell.2018.00171

**Published:** 2019-01-14

**Authors:** Marius Volker Dannappel, Dhanya Sooraj, Jia Jian Loh, Ron Firestein

**Affiliations:** ^1^Hudson Institute of Medical Research, Clayton, VIC, Australia; ^2^Department of Molecular and Translational Sciences, Monash University, Clayton, VIC, Australia; ^3^Faculty of Science, School of Biological Sciences, Monash University, Clayton, VIC, Australia

**Keywords:** mediator kinase, cyclin-depedent kinase, Cdk8, CDK19, developmental signaling, mouse models, development, tissue homeostasis

## Abstract

CDK8 and its paralog, CDK19, collectively termed ‘Mediator Kinase,’ are cyclin-dependent kinases that have been implicated as key rheostats in cellular homeostasis and developmental programming. CDK8 and CDK19 are incorporated, in a mutually exclusive manner, as part of a 4-protein complex called the Mediator kinase module. This module reversibly associates with the Mediator, a 26 subunit protein complex that regulates RNA Polymerase II mediated gene expression. As part of this complex, the Mediator kinases have been implicated in diverse process such as developmental signaling, metabolic homeostasis and in innate immunity. In recent years, dysregulation of Mediator kinase module proteins, including CDK8/19, has been implicated in the development of different human diseases, and in particular cancer. This has led to intense efforts to understand how CDK8/19 regulate diverse biological outputs and develop Mediator kinase inhibitors that can be exploited therapeutically. Herein, we review both context and function of the Mediator kinases at a molecular, cellular and animal level. In so doing, we illuminate emerging concepts underpinning Mediator kinase biology and highlight certain aspects that remain unsolved.

## Introduction

Transcription, the first stage of gene expression, is a fundamental cellular process by which genetic information (DNA) is converted to a molecule (RNA) that can exert a molecular phenotype. Aberrant transcription has been implicated as a causal event in diverse human diseases, underscoring the need to better understand and control this critical process. In recent years a slew of small molecule inhibitors have been developed and utilized to target proteins that control transcription at the either the epigenetic (Dokmanovic et al., 2007; Belkina and Denis, 2012; Shi and Vakoc, 2014; West and Johnstone, 2014) or genetic level (Bensaude, 2011). Specifically in cancer, where extraordinarily high levels of transcription are required to drive oncogenic output, inhibitors of general transcription, have shown promising activity in preclinical studies, and early phase clinical trials. These early successes underscore the need to better understand the regulatory networks that underlie transcriptional control.

At the heart of the basal transcriptional machinery is the holoenzyme RNA polymerase II (RNPII), composed of 12 subunits that mediate the transcription of DNA to messenger RNA (mRNA). RNPII, in turn, is regulated by a macromolecular complex called the pre-initiation complex (PIC), which consists of TFIIA, TFIIB, TFIIE, TFIIF, TFIID, TFIIH, SAGA, P-TEFb, and Mediator (Nikolov and Burley, 1997; Thomas and Chiang, 2006). A central aspect of RNPII regulation, involves the ability of distinct kinases within the PIC complex to modulate RNPII activity either directly (RNPII phosphorylation) or indirectly. One of the most highly conserved function of these kinases is the phosphorylation of the C-terminal domain (CTD) of RNPII, a key event in regulating transcription initiation, elongation, and RNA processing (Poss et al., 2013; Allen and Taatjes, 2015). This process is highly conserved from yeast to human and involves three distinct members of the cyclin dependent kinase family, CDK7, CDK8, and CDK9.

Cyclin-dependent kinases (CDKs) are serine/threonine protein kinases that serve as key regulators of both cell cycle and transcription. CDKs require the binding of a cyclin subunit to exert their enzymatic activity and are broadly divided into two functional subgroups, cell cycle CDKs, and transcriptional CDKs (Malumbres, 2014). Among these transcriptional CDKs, CDK7, CDK8, and CDK9 are the most highly studied. While each transcriptional CDK binds to its cognate cyclin and can phosphorylate the CTD of RNPII, CDK8 has emerged as a unique member of this subclass, based on its ability to fine-tune transcriptional output by not only phosphorylating RNPII itself, but also associated sequence specific transcription factors (Alarcón et al., 2009; Bancerek et al., 2013). To do so, CDK8 reversibly interacts with the Mediator complex, as part of a four subunit complex termed the CDK8 module. This module consists of CDK8, its cognate cyclin (CCNC), Mediator complex subunit 12 (MED12) and Mediator complex subunit 13 (MED13) (Allen and Taatjes, 2015). The kinase activity of CDK8 has been intimately linked to its ability to bind both CCNC and MED12, highlighting the importance of submodule integrity for CDK8 effector functions (Knuesel et al., 2009a,b). As part of this complex, CDK8 has been shown to regulate transcription factor phosphorylation, activity, and turn-over, thus impacting key transcriptional outputs affecting cellular and developmental homeostasis (Fryer et al., 2004; Alarcón et al., 2009; Adler et al., 2012; Bancerek et al., 2013; Poss et al., 2013). In recent years, an alternative Mediator kinase module consisting of genes with high homology to CDK8, MED12, MED13 have been described in vertebrates. The CDK8 homolog, CDK19 (Cdk11, CDC2L6) was discovered by Hidden Markov model (HMM) profiling of a eukaryotic protein kinase domain, and found to have approximately 91% sequence homology to Cdk8. The two proteins share a particularly high degree of sequence conservation in their kinase and cyclin binding domain (Manning et al., 2002), however CDK8 and CDK19 diverge at their C-terminal tails suggesting the two proteins may regulate different interaction partners. Consistent with this, CDK19 forms a Mediator kinase module distinct to the CDK8 module and has been found to regulate different transcriptional programs (Daniels et al., 2013; Galbraith et al., 2013). In this review, we describe the roles of the two Mediator kinases CDK8 and CDK19 in both normal tissue homeostasis and disease states. In so doing, we highlight the complex nature of transcriptional regulation in signaling pathways and cellular responses that are implicated in both development and disease.

## The CDK8 Kinase Module in Yeast

Components of the Mediator kinase module are highly conserved and were initially identified in *Saccharomyces cerevisiae* (yeast) as transcriptional regulators of the RNPII holoenzyme (Hengartner et al., 1995; Kuchin et al., 1995; Liao et al., 1995). In yeast, CDK8 is essential for the regulation of genes involved in cell type specificity, meiosis and sugar utilization, but is not required generally for gene expression as CDK8 mutant cells express large subsets of genes normally. Deletion of CDK8 or CyclinC (CCNC) in *S. cerevisiae* rescued the growth defect resulting from truncation of the RNA polymerase II carboxy-terminal domain (CTD), which has been associated with impaired gene expression (Surosky et al., 1994; Kuchin et al., 1995; Liao et al., 1995; Wahi and Johnson, 1995). The CDK8/CCNC pair phosphorylates the CTD of RNPII *in vitro* and this phosphorylation occurs before PIC formation to inhibit transcriptional initiation (Liao et al., 1995; Hengartner et al., 1998). In contrast to the deletion or mutations of core Mediator components, which resulted in reduced viability, deletion of the Mediator kinase module subunits MED12, MED13, CDK8 and CCNC did not affect cell viability, but caused flocculation and mild temperature sensitivity in *S. cerevisiae*. Microarray analysis using a kinase dead CDK8 mutant revealed upregulation of approximately 3% of all genes in *S. cerevisiae* (Koleske et al., 1992; Thompson et al., 1993; Hengartner et al., 1995; Liao et al., 1995; Holstege et al., 1998). Altogether, these results contributed to the classification of CDK8 and CCNC as a transcriptional CDK-cyclin pair and more specifically, as a negative regulator of a specific subset of genes acting within the Mediator complex.

Further studies have unraveled new regulatory mechanisms by which CDK8 controls transcription in different cellular settings or in response to environmental stress-signals. A comprehensive study, systematically assessing the effects of individual deletion of all 15 non-essential Mediator subunits on global transcription in *S. cerevisiae*, identified three classes of Mediator subunits based on distinct expression profiles (van de Peppel et al., 2005). In line with previous studies, CDK8 kinase module (CKM) subunits were identified as negative transcriptional regulators, as opposed to other Mediator components MED2 and MED18 which were associated with positive transcriptional output (van de Peppel et al., 2005). Further, the authors could demonstrate that CCNC/CDK8 are upstream of MED2 and negatively regulate it by phosphorylation which repressed the activation of the low-iron response transcription factor Rcs1/Atf1, establishing a new antagonistic mechanism of CDK8-mediated transcriptional regulation via phosphorylation of Mediator subunits (van de Peppel et al., 2005).

Beside its role in transcriptional repression, the CKM has been implicated with positive regulation of gene expression under defined stress conditions. For instance, Sip4 is a transcription activator repressed in the presence of glucose, but upregulated in response to glucose limitation and able to bind to carbon source-responsive elements in gluconeogenic genes. CDK8 was shown to directly interact with- and phosphorylate Sip4. Phosphorylation of Sip4 by CDK8 positively correlated with the expression of a LexA-Sip4 *lacZ* reporter, establishing CDK8 as a positive regulator of gene expression during growth in carbon sources other than glucose (Vincent et al., 2001).

The presence of a fermentable carbon source and the absence of nitrogen initiate a sporulation signal in yeast that can induce meiosis. In CCNC null strains, the first meiotic division (meiosis I) was delayed or completely skipped in more than half of all cells, resulting in formation of two diploid spores (dyads). These results suggest an important role for CCNC during meiosis in yeast (Cooper and Strich, 2002). Inducer of meiosis 1 (IME1) is a master transcription factor regulating expression of genes important for meiosis in yeast. Induction of IME1 was delayed in CCNC-deficient strains in sporulation media, although the maximal expression levels were similar between wildtype and CCNC null strains (Cooper and Strich, 2002). Similarily, CDK8 has been shown to be essential for maximal induction of IME1, as IME1 expression was decreased during meiosis in CDK8-deleted strains (Ohkuni and Yamashita, 2000). Although the molecular mechanism by which CDK8-CCNC drives meiotic gene expression program remains elusive, these results demonstrate that CDK8-CCNC also activate transcription to regulate a developmental program in yeast.

The same growth conditions (nitrogen limitation and the presence of a fermentable carbon source) can induce pseudohyphal growth, a differentiation pathway in yeast which phenotypic occurrence correlates with the expression of FLO11 (Law and Ciccaglione, 2015). During rich growth conditions, pseudohyphal growth is inhibited in a process regulated by CDK8 and CCNC (Law and Ciccaglione, 2015). Upon combinatorial deletion of CCNC or CDK8 together with JHD2, a histone demethylase, FLO11 was constitutively expressed and double mutant cells showed a pseudohyphal budding pattern (Law and Ciccaglione, 2015). Interestingly, CCNC-JHD2 double knockout cells showed increased Histone3 Lys4 trimethylation in the promoter and coding region of FLO11, facilitating constitutive transcription of FLO11. This observation was locus-specific, as these deletions did not affect trimethylation of other control loci (NUP85, 18S rDNA).

Although the exact mechanism of how CDK8-CCNC represses H3 Lys4 trimethylation at the FLO11 locus is unknown, it has been suggested that CDK8/CCNC might interact with the transcriptional repressor Sfl1p to inhibit gene expression at the target locus as it has been described for SUC2 (Song and Carlson, 1998). Further studies are required to unravel the mechanism by which CDK8-CCNC can regulate the methylation of promoter- and coding regions. Collectively, these studies demonstrate a role for mediator kinases in controlling differentiation (upon environmental stress) and cell-fate decisions in yeast that extend beyond general transcriptional output.

## CDK8 Kinase Module in *Drosophila* Development

Orthologs of the Mediator and the Mediator kinase module subunits are found in metazoans, however their sequences have diverged during evolution, while retaining a high degree of homology in the kinase domain (Bourbon, 2008; Poss et al., 2013). Genetic and biochemical experiments in metazoan cells demonstrated that the CDK8/CCNC/MED12/MED13 module reversibly associates with the Mediator and shares an overall structural organization with the yeast mediator kinase module (Wang et al., 2001; Taatjes et al., 2002; Tsai et al., 2013, 2014). Evidence for functional diversification during evolution was obtained from genome wide targeting of CKM subunits. In *Drosophila melanogaster*, all four kinase module subunits CDK8, CCNC, MED12, and MED13 are essential for development (Treisman, 2001; Janody et al., 2003; Loncle et al., 2007). Homozygous CDK8 and CCNC mutant animals died at late third-instar larvae (L3/early pupae), whereas homozygous MED12 and MED13 mutants die as late embryos/early first-instar larvae (Loncle et al., 2007). In addition, MED12 and MED13 are required for early eye development by regulating *dac* and *dpp* expression independently of CDK8 and CCNC, whereas all kinase module subunits function together in external sensory development (Loncle et al., 2007). These results demonstrate an essential function for the CDK8-kinase module during development, but also suggest a kinase-independent function of MED12/MED13 in regulating gene expression. MED12 has been shown to interact with different transcriptional regulators and thus, deletion of MED12 possibly affects the interaction of transcription factors necessary for development with the core Mediator/ RNPII. We speculate that impaired interaction could account for the unique phenotypic consequences of MED12- and MED13-deficient embryos (Kim et al., 2006; Zhou et al., 2006; Loncle et al., 2007; Poss et al., 2013).

## CKM in Development

In mice and humans, CDK8 is expressed ubiquitously and has been implicated in different physiologically processes (Tsutsui et al., 2008; Galbraith et al., 2013; McCleland et al., 2015; Thul et al., 2017; Uhlén et al., 2015; Uhlen et al., 2017). Inactivation of CDK8 in mice by insertion of a gene trap deleting the kinase domain of CDK8 resulted in embryonic lethality at E2.5-3 before preimplantation of the *Cdk8^-/-^* embryos, indicating a role of CDK8 before or during the 8-cell stage (Westerling et al., 2007). Although the defined role of CDK8 during early embryonal development remains elusive in this model, in line with the reported functions as a transcriptional regulator, it is possible that lethality of *Cdk8^-/-^* embryos is due to transcriptional de-regulation. In line with this, another study revealed a role for CDK8 in maintaining murine embryonic stem cells in an undifferentiated state (Adler et al., 2012). shRNA-mediated knockdown of CDK8 resulted in reduced levels of transcription factors MYC, OCT4 and NANOG, all important to maintain proliferative capacity and pluripotent state of embryonic stem cells (Young, 2011; Adler et al., 2012). Genome-wide chromatin immunoprecipitation experiments in CDK8-knockdown ES cells revealed enrichment of MYC stem cell target genes among the CDK8-induced genes, suggesting that CDK8 promotes pluripotency possibly by regulating MYC target gene expression. CDK8-knockdown increased MYC-Threonine-58 phosphorylation, which marks it for degradation, showing that CDK8 controls stability of MYC post-translationally (Adler et al., 2012). It remains unknown whether CDK8 controls MYC phosphorylation directly or indirectly, however MYC has not been reported to be a substrate for CDK8 in human colon cancer cells (Poss et al., 2016). It would be interesting to test whether the ability of CDK8 to regulate the pluripotency transcription factors accounts for the early embryonic lethality seen in *Cdk8^-/-^* mice.

Genetic disruption of the other Mediator kinase module subunits, has also been found to result in embryonic lethality, cementing the importance of this complex in early embryogenesis (Rocha et al., 2010; Li et al., 2014; Miao et al., 2018). *Ccnc^-/-^* mice die *in utero* at E10.5 and exhibited severe developmental retardation and an underdeveloped placental labyrinth layer (Li et al., 2014). As expected, CDK8 kinase activity is lost in CCNC-deficient murine embryonic fibroblasts (MEFs), and CDK8 was not able to interact with MED12 and MED13 demonstrating that CCNC is essential for the formation of the CDK8 module (Li et al., 2014). Interestingly, endogenous CTD phosphorylation was not affected, suggesting the presence of another, yet to be identified kinase, being able to phosphorylate CTD *in vivo* (Li et al., 2014). Gene expression analysis in *Ccnc^-/-^* MEFs and brains showed no major differences compared to wildtype controls. Conditional ablation of CCNC in haematopoietic cells revealed a role for CCNC-CDK3, CCNC-CDK8, and CCNC-CDK19 in the negative regulation of Notch signaling. Notch signaling controls cell fate decisions in many organs by releasing Notch receptor intracellular domain (ICN1) which forms a complex with cofactors CBF1 and MAML to activate the response genes (Fryer et al., 2004; Li et al., 2014). CDK8 and CCNC have been shown to negatively regulate Notch signaling by interacting with Mastermind (MAM) to form a CCNC:CDK8 Notch destruction complex that localizes to subnuclear foci. Within this complex, CDK8 phosphorylates ICN1 targeting it for degradation and thus, coordinates Notch signaling by controlling ICN1 turnover (Fryer et al., 2004). This regulatory function of CCNC-CDK8-mediated Notch turnover was shown to be important *in vivo* to limit differentiation of bone marrow cells toward the T-cell lineages (Li et al., 2014). Consequently, CCNC ablation but also Cyclin C heterozygosity resulted in elevated ICN1 levels and accelerated T-cell acute lymphoblastic leukaemia (T-ALL) identifying CCNC as a haplosufficient tumor suppressor (Li et al., 2014).

Knockdown and conditional knockout of MED13 revealed an essential function for MED13 during mouse zygotic genome activation. MED13 interacts with E2F transcriptions factors to direct expression of zygotic genes, among them esBAF, an embryo-specific chromatin remodeling complex, which in turn facilitates reprogramming of the zygote to a totipotent embryo (Miao et al., 2018). MED13L knockdown embryos die before preimplantation of the embryo, similar to MED13 knockout embryos. Importantly, MED13L partially compensated the loss of MED13 but fails to compensate post-implantation development, showing that first, MED13L is required for embryonic development and second, MED13 and MED13L seem to be partly redundant but also control clearly distinct developmental programs (Miao et al., 2018). In addition, MED13 and MED13L are important role during neuronal development. Genetic alterations leading to a truncated version of MED13 or Med13 harboring missense mutations have been found in humans with intellectual disability and/or developmental- and speech delays. Likewise, MED13 mutations are associated with autism spectrum disorder and optic nerve abnormalities (Snijders Blok et al., 2018). Similarily, several heterozygous loss-of-functions mutations for MED13L have been reported resulting in intellectual disability and a distinctive facial appearance in affected people (Asadollahi et al., 2013; Cafiero et al., 2015; van Haelst et al., 2015). These results demonstrate that MED13L haplosufficiency results in mental disability, underscoring an important role for the CKM in neuronal development beyond the zygote stage.

MED12 binds indirectly to CDK8 via CCNC and is critical for CDK8 kinase activity (Knuesel et al., 2009b). Consistent with this, expression of a hypomorphic MED12, reducing MED12 protein levels by more than 90%, results in embryonic lethality at E10.5 due to neural tube closure defects, cardiac malformations, body axis truncation, and defective somitogenesis (Rocha et al., 2010). Mice with complete ablation of MED12 die at E7.5 because they fail to form a primitive streak and the distal visceral endoderm does not initiate migration to form the anterior visceral endoderm (Rocha et al., 2010). β–catenin-deficient embryos exhibit a similar phenotype as MED12-deficient embryos, and in both cases, the expression of Wnt/β–catenin target genes is impaired, establishing MED12 as an essential mediator of Wnt/β–catenin signaling for anterior posterior formation during embryogenesis (Huelsken et al., 2000; Rocha et al., 2010). MED12 was identified as a key regulator of β-catenin mediated transcription that directly binds to β-catenin. This interaction is required to direct the Mediator complex to Wnt-responsive genes (Kim et al., 2006). Our team and others has specifically implicated CDK8 as a transducer of β-catenin mediated transcription. An integrated genomic analysis of colon cancer showed that CDK8, itself, is amplified in colorectal cancer and is important for mediating Wnt/β-catenin signaling in a subset of cancers where it is overexpressed or amplified (Firestein et al., 2008). As CDK8 and MED12 have both been implicated in mediating Wnt/β–catenin signaling, it would be interesting to speculate whether MED12 exerts its function within the mediator in a CDK8-dependent manner or if MED12 and CDK8 control Wnt/β–catenin signaling at different contextual and temporal levels.

While CDK8 is required for early embryogenesis, preliminary analysis of CDK19 knockout mice by the International Mouse Phenotyping Consortium (IMPC) reported no significant abnormalities. These mice are born at expected Mendelian ratios and have a body weight as well as a normal life-span. Analysis performed by the IMPC did not detect behavioral differences and no phenotypes in analyzed compartments such as the neurological-, reproductive- or cardiovascular-system. Thus, CDK19 is dispensable for both embryonic development and adult tissue homeostasis (Dickinson et al., 2017). CDK19-deficient primary cells (e.g., MEFs) have not been published yet, demonstrating the urge of further studies to unravel the cellular functions of CDK19 in non-transformed cells.

Altogether, these studies demonstrate essential functions of the mediator kinase subunits for vertebrate embryogenesis and functional diversification during evolution. The differences observations made in *Cdk8^-/-^* and *Cdk19^-/-^* mice demonstrate clearly distinct functions of CDK8 and its homolog CDK19. In some specific instances, CKM subunit members might have a certain degree of functional redundancy. For instance, MED12L can bind Sox10 during myelination in a similar way to MED12 and both, CDK8 and CDK19 can interact with the histone arginine methyl transferase PRMT5 to repress transcription (Tsutsui et al., 2013; Vogl et al., 2013). However, despite MED13 and MED13L seem to be partly redundant *in vivo* during development, evidence for functional redundancy is missing (Miao et al., 2018).

## The Mediator Kinase Submodule in Adult Tissue Homeostasis

In contrast to its role during embryonic development and developmental signaling pathways, the function of CDK8 and its interaction partners in adult tissue homeostasis are less well studied. In cardiomyocytes, MED13 controls transcription of nuclear hormone receptors and is negatively regulated by microRNA miR-208a (Grueter et al., 2012). Overexpression of MED13 in cardiomyocytes protected mice from high-fat diet-induced obesity and improved glucose tolerance and insulin sensitivity. In contrast, cardiac-specific deletion of MED13 increases obesity upon high-fat diet and susceptibility to metabolic syndrome (Grueter et al., 2012). Mechanistically, MED13 increases energy expenditure and controls expression of genes involved in metabolism and thus, is important for the regulation of systemic energy homeostasis (Grueter et al., 2012). In line with these results, MED13, but also MED12 have been shown to control obesity by regulating Wingless in *Drosophila* (Lee et al., 2014).

MED12 is essential for maintaining haematopoietic stem cell (HSC) homeostasis, as induced deletion of MED12 in HSCs and progenitor cells (HSPCs) causes bone marrow aplasia resulting in lethality within 4 days (Aranda-Orgilles et al., 2016). Analysis in human HPSCs could show that the activity of haematopoietic transcription factors at enhancer regions is coordinated by MED12 along with H3K27 acetylation at enhancer regions to promote transcriptional activation. Surprisingly, this function of MED12 is independent of Mediator kinase module, as deletion or knockdown of CDK8, CCNC, or MED13 did not affect the HPSC colony-forming capacity. The whole pool of cellular MED12 was associated with the Mediator complex and no free MED12 observed, suggesting that MED12 acts within the core Mediator complex in a Mediator kinase module-independent manner (Aranda-Orgilles et al., 2016).

Tamoxifen-inducible ubiquitous deletion of CDK8 in adult mice is compatible with animal health and does not result in an overt phenotype, indicating that in contrast to CCNC and MED12, CDK8 is not required for adult tissue homeostasis. In line with this, intestinal epithelial cell specific ablation of CDK8 does not impair intestinal homeostasis (McCleland et al., 2015). Surprisingly, in the *Apc^Min^* model, a murine model of colorectal cancer (CRC), CDK8 deletion shortens the survival of the mice and increasing tumor burden. While tumor initiation is not affected, tumor growth rate and tumor size are increased upon ablation of CDK8, accompanied by reduced histone H3K27 tri-methylation affecting the promoters of PcG-regulated genes and increasing oncogenic signaling (McCleland et al., 2015). Thus, in addition to its role as a CRC oncogene in driving Wnt/β–catenin signaling in invasive colon cancers, CDK8 acts as a tumor suppressor presumably in early stages of intestinal tumorigenesis (Meijer et al., 1998; Firestein et al., 2008; McCleland et al., 2015).

To investigate the function of CDK8/19 kinase activity *in vivo* and assess their therapeutic potential, two structurally distinct CDK8/19 inhibitors were developed and tested in mice. The two compounds were tolerated well by mice with sporadic cases of body weight loss, suggesting that kinase activity of CDK8 and CDK19 is dispensable for adult homeostasis (Clarke et al., 2016). The same CDK8/19 inhibitors injected into rats and dogs resulted in adverse effects in multiple tissues, including dysplasia of the growth plate, decrease in the proliferative zone and increased proliferation of irregular woven bone in the bone cavity. Additionally, apoptotic lesions were observed in the pancreas, intestinal epithelium, male reproductive tract, hair follicles, heart, and lymphatic tissues in rats. In contrast, proliferative lesions occurred in the lungs, liver, thymus, heart, mammary gland and male reproductive system (Clarke et al., 2016). These results may be explained with a further functional diversification CDK8/19 from mice to rats and dogs during evolution, which may restrict the use CDK8/19 kinase inhibitors in the clinics. This highlights the complexity of Mediator kinase activity *in vivo* and imply that the physiological roles of CDK8 and CDK19 need to be carefully assessed in the appropriate context and model.

## Mediator Kinase Function in Response to Hypoxia

Hypoxia occurs in growing tissues such as embryos and solid tumors and requires a transcriptional response initiated by hypoxia-inducible factors (HIFs) to adapt to hypoxic conditions. CDK8 but not CDK19 is required for HIF1A induced gene expression upon hypoxia in CRC cells (Galbraith et al., 2013; Chen and Lou, 2017). Mechanistically, CDK8 co-activates HIF1 target genes by recruiting Super Elongation complex (SEC) and positive transcription elongation factor b (P-TEFb) resulting in the release of paused RNPII and RNPII elongation (Galbraith et al., 2013).

As an adaptation to intermittent hypoxia and to meet their energy demand, cancer cells re-program their energy metabolism and upregulate glycolytic enzymes, usually induced by HIF1A. Recent studies utilizing bio-orthogonal approach to explore the role of Cdk8 kinase in glycolysis revealed that impaired kinase activity reduced glycolysis and sensitized colon cancer cells to the glucose analog 2- deoxy - D glucose (Galbraith et al., 2017). Colorectal cancer cells expressing an ATP analog sensitivity CDK8 variant (CDK8- AS) exhibited growth defects and anti-tumorigenic properties both *in vitro* and *in vivo* in the presence of ATP analog 3MB-PP1. In these cells, CDK8 kinase activity was required for maximum expression of ∼7.5% of all genes during normoxia, and the maximum expression of ∼17% of all genes during hypoxia and 23.3% of hypoxia inducible genes is CDK8-kinase activity dependent (Galbraith et al., 2017). Among the CDK8 kinase-dependent regulated genes, hypoxia and glycolysis gene sets were significantly enriched under both hypoxic and normoxic conditions. CKD8-dependent gene expression of glycolytic genes was shown to be conserved among 25 different cancer cell lines and independent of CDK19 (Galbraith et al., 2017). Collectively these findings imply that mediator associated kinases CDK8 and its paralog CDK19 have functionally distinct roles in regulating gene expression programs in metabolic processes. It is worth noting that these studies have been performed in transformed cells with an altered metabolic activity that adapted to the culturing conditions compared to primary cells. It remains to be seen if CDK8 and CDK19 regulate hypoxia during development and tumor growth *in vivo*.

## Mediator Kinase in Response to Dna Damage and Chemotherapy

Eukaryotic cells are constantly exposed to stress/ insults, which lead to the accumulation of genomic instability. DNA Damage Response (DDR) pathways, activated upon sensing of single- and double strand breaks by ATR and ATM, prevent accumulation of damaged DNA and thus, maintain genomic stability of cells (Jackson and Bartek, 2010; Yan et al., 2014). Recent pan cancer analyses using The Cancer Genome Atlas (TCGA) across 23 cancer types, followed by tissue microarray (TMA) expression validation, revealed high CDK19 expression in primary prostate tumors and high expression of both, CDK8 and CDK19 in prostate cancer metastases (Brägelmann et al., 2017). The use of a highly specific dual CDK8/19 kinase inhibitor in prostate cancer cells induced ATR-mediated DDR resulting in caspase-independent cell death (Nakamura et al., 2018).

p53 (*Trp53*) is a well know tumor suppressor that regulates various cellular processes such as DNA repair, cell cycle and apoptosis upon DNA damage to prevent accumulation of mutations in the genome. In osteosarcoma cells that do not express CDK8, knockdown of CDK19 resulted in reduced proliferation, accompanied by reduced mitotic gene expression. In contrast, genes involved in the p53 pathway and cholesterol metabolism were upregulated (Audetat et al., 2017). Treatment of 5-fluorouracil (5-FU) to activate genotoxic stress, resulted in a 2-fold reduction of differentially regulated genes between CDK19 knockdown and control cells. Interestingly, genes induced in CDK19 knockdown cells were distinct from those induced in control cells. Nutlin-3, an inhibitor of the negative p53 regulator murine double minute 2 homolog (MDM2), resulted in p53 stabilization and subsequent p53 target gene induction. In Cdk19 knockdown cells, expression of p53 target genes *Cdkn1a* (p21) and *Bbc3* (PUMA) in response to Nutlin-3 was reduced. As PUMA is a pro-apoptotic protein, reduced levels of apoptosis were observed (Audetat et al., 2017). These results were obtained in a very defined setting (osteosarcoma cell lines which does not express CDK8) and the mechanism by which CDK19 regulates p53 target gene expression remains elusive. In colon cancer cells, CDK8 was recruited to the p21 locus upon transcriptional activation in a stimulus-specific manner (Donner et al., 2010). This raises the question whether CDK8 and CDK19 have a more general role in the DNA damage response and if this is important *in vivo* to prevent the formation of tumors in tissues exposed to DNA damaging agents (such as skin UV radiation).

CDK8/19 have also been implicated in modulating the paracrine activity of chemotherapy. Chemo-radiotherapy elevates tumor inducing paracrine effects, which ultimately leads to treatment resistance and secretion of multiple tumor promoting cytokines. Treatment of colon cancer cells with doxorubicin induced paracrine anti-apoptotic activity in an *in vitro* co-culture system (Porter et al., 2012). A dual CDK8/19 inhibitor, Senexin-A, was found to inhibit p21 transcription, and reversed paracrine associated anti-apoptosis both *in vitro* and *in vivo*. Further, meta-analysis of gene expression data on breast and ovarian cancer showed that high expression of both CDK8 and 19 strongly correlated with poor survival rate in patients treated with DNA damaging agents such as platinum-based chemotherapy (Porter et al., 2012). Altogether, these findings suggest a cognate role of CDK8 and CDK19 in cellular DNA damage responses, however these studies need to be further tested *in vivo*.

## Inflammation and Innate Immune Response

The Nuclear factor- κB (NF-κB) family of transcription factors are master-regulators of inflammation and have been implicated in cancer (Ben-Neriah and Karin, 2011). Recently, CDK8/19 have been shown to regulate NF-κB-mediated gene expression in response to different stimuli. Upon stimulation with Tumor Necrosis Factor-α (TNF- α), NF-κB heterodimers and CDK8/19 were co-recruited to the promoters and drive expression of NF-κB early response genes, *Il8*, *Cxcl2*, and *Cxcl3*. Inhibition of CDK8/19 kinase activity suppressed expression of selected NF-κB target genes, but did not inhibit basal expression of NF-κB-regulated genes (Chen et al., 2017). During Toll like receptors (TLRs)-induced gene expression, both CDK8 and 19 together with NF-κB and C/EBPβ were shown to co-localize at the promoter region of inflammation associated genes such as *Il8*, *Il10*, *Ptx3*, and *Ccl2* to positively regulate the transcription of these genes (Yamamoto et al., 2017). These studies directly implicate the CKM in molecular pathways critical for the regulation of inflammatory gene expression, proper clearance of infections and inflammatory checkpoints.

Interferons (IFNs) are signaling proteins that provide a first unspecific line of defense against invading pathogens (Schneider et al., 2014). CDK8 was shown to phosphorylate STAT1, STAT3 and STAT5, transcription factors activated by Janus-activated kinase (JAK) downstream of interferon receptors, within their transactivation domains (TADs) upon IFN-γ stimulation (Bancerek et al., 2013) Over 40% of all IFN-γ responsive genes were positively or negatively regulated by CDK8-mediated phosphorylation of STAT1, thus identifying CDK8 as an important regulator of antiviral responses (Bancerek et al., 2013). Two different studies using CDK8/19 inhibitors Cpd32 and Cortistatin A showed reduced levels of phosphorylated STAT1 in response of IFN-γ stimulation, confirming a kinase-dependent function of CDK8/19 to regulate antiviral gene expression (Pelish et al., 2015; Koehler et al., 2016).

Interestingly, there is evidence that CDK8/19 might regulate cytokine expression *ex vivo*. Tissue co-culture models using different primary cells revealed a modulated expression of various cytokines such as IL-8 and IL-17A, IL-17F upon CDK8/19 inhibition, suggesting that CDK8/19 may have a function in inflammatory gene expression *in vivo* (Clarke et al., 2016). While CDK8/19 dysregulation or mutations have not been associated with chronic inflammatory diseases or susceptibility upon bacterial and viral infections, it would be interesting to check whether abnormal CDK8 or CDK19 activity contribute in pathology in patients with chronic inflammatory diseases such as inflammatory bowel disease (IBD).

## Conclusion

CDK8 and CDK19 are among a unique class of cyclin dependent kinases with non-cell cycle roles. In this review, we have summarized the molecular interaction of these kinases with Mediator, their pleiotropic effects on multiple signaling pathways and their important roles in both embryonic development and adult tissue homoestasis. An overarching theme from these studies is the exemplary ability of these kinases to impinge on different pathways in a context specific manner. Future studies focusing on the epigenetic and molecular contexts that underpin CKM regulatory effects will be critical to advance our understanding of how gene expression can be fine tuned in a temporal and spatial manner.

## Author Contributions

RF designed the topic of the review. MD, DS, and JL wrote the manuscript. MD and RF edited the manuscript. RF approved the final version.

## Conflict of Interest Statement

The authors declare that the research was conducted in the absence of any commercial or financial relationships that could be construed as a potential conflict of interest.
